# Comparison of Selenium-Enriched *Lactobacillus* *paracasei*, Selenium-Enriched Yeast, and Selenite for the Alleviation of DSS-Induced Colitis in Mice

**DOI:** 10.3390/nu14122433

**Published:** 2022-06-12

**Authors:** Yuqing Zhong, Yan Jin, Qiuxiang Zhang, Bingyong Mao, Xin Tang, Jie Huang, Renmei Guo, Jianxin Zhao, Shumao Cui, Wei Chen

**Affiliations:** 1 State Key Laboratory of Food Science and Technology, Jiangnan University, Wuxi 214122, China; 6200113147@stu.jiangnan.edu.cn (Y.Z.); zhangqx@jiangnan.edu.cn (Q.Z.); maobingyong@jiangnan.edu.cn (B.M.); xintang@jiangnan.edu.cn (X.T.); zhaojianxin@jiangnan.edu.cn (J.Z.); chenwei66@jiangnan.edu.cn (W.C.); 2 School of Food Science and Technology, Jiangnan University, Wuxi 214122, China; 3 The Affiliated Wuxi Second People’s Hospital of Nanjing Medical University, Wuxi 214002, China; 13951514799@163.com; 4 Suzhou Setech Biotechnology Co., Ltd., Suzhou 215000, China; jhuang@justug.com (J.H.); rmguo@justug.com (R.G.); 5 National Engineering Research Center for Functional Food, Jiangnan University, Wuxi 214122, China

**Keywords:** Se forms, colitis, Se supplement, Se-enriched *Lactobacillus paracasei*, selenocysteine

## Abstract

Patients with inflammatory bowel disease (IBD) have been found to have decreased immune function. Selenium (Se) is an essential trace element that is beneficial for human health, which has a significant stimulating effect on immune function. We compared the effects of different Se forms on the alleviation of colitis in DSS-induced mice. Moreover, we also aimed to determine whether Se-enriched *Lactobacillus paracasei* CCFM 1089 could be used as a new organic Se supplement. Different Se supplements (Se-enriched *L. paracasei* CCFM 1089, Se-enriched yeast and sodium selenite) were given to Se-deficient mice suffering from colitis. Se-enriched *L. paracasei* CCFM 1089, which is based on selenocysteine (SeCys), had similar effects in terms of reducing oxidative stress and inhibiting pro-inflammatory factors to Se-enriched yeast; however, selenase activity in the Se-enriched *L. paracasei* CCFM 1089-treated mice was higher than that in other treatment groups. In addition, Se-enriched *L. paracasei* CCFM 1089 could better protect the intestinal mucosa, which increased the expression of tight junction proteins (ZO-1 and occludin) in mice. Thus Se-enriched *L. paracasei* CCFM 1089 was shown to alleviate IBD, suggesting that it has potential as a good organic Se supplement.

## 1. Introduction

Inflammatory bowel diseases (IBDs), including Crohn’s disease and ulcerative colitis (UC) [1], are common autoimmune chronic diseases [2,3] that are difficult to cure and recur easily. UC can cause ulcerations and mucosal congestion in colonic lesions, diarrhea, and blood release in the stool [4]. Although the prevalence of IBD has increased in recent years, its pathogenesis has not been completely explored. Oxidative stress, intestinal barrier damage, and other factors are speculated to be related to the pathogenesis of IBD [5]. At present, amino salicylic acid, antibiotics, and adrenocorticosteroids are the main drugs used to treat patients with IBD. Long-term drug use can cause drug dependence or induce anemia [6]. Therefore, new methods must be developed for the treatment of IBD.

Clinical studies have found that selenium (Se) levels in the serum of patients with IBD are significantly reduced compared to those in healthy people (0.19 vs. 0.93, *p* < 0.05) [7]. With several weeks of sodium selenite supplementation, IBD symptoms, such as the increase in macrophages and inflammatory factors, improve [8]. Shi et al. [9] found that the oral administration of Se-containing amino acids to mice with colitis alleviates IBD by reducing oxidative stress and intestinal inflammation.

Se is an essential trace element, beneficial for human health [10,11]. It exists in two forms, inorganic and organic, with the following bioavailabilities: 25.0–49.9% for inorganic Se and 75.7–89.5% for organic Se [12]. The main inorganic forms are selenate and selenite. A high dose of inorganic Se is highly toxic and can accelerate oxidation in the body. Organic Se is mainly composed of selenoproteins and Se-containing amino acids such as selenomethionine (SeMet) and selenocysteine (SeCys). This plays a key role in maintaining biological functions. The production process of synthetic organic Se is complicated; it cannot be mass-produced at present. The established organic Se products currently available on the market are Se-enriched yeast powder and Se-enriched yeast tablets. Se-enriched yeast has been shown to be a superior Se supplement [13].

As only one type of organic Se supplement is currently available, it is necessary to develop new organic Se products. Previous studies have shown that probiotics can be introduced to medium containing inorganic Se using the biotransformation method, which can convert inorganic Se into organic Se and form Se-enriched probiotics [14]. SeMet and SeCys are the main forms of Se in Se-enriched products. The main form of Se in Se-enriched yeast is SeMet [15]. Notably, SeCys is the 21st amino acid discovered in recent years. As a third-generation organic Se compound, SeCys is the active center of selenoproteins [16,17,18,19] and drives them to perform physiological functions. Therefore, we assumed that SeCys-enriched products would perform better than SeMet-enriched products. To verify this conjecture, we selected a strain with a strong SeCys-enriched ability.

In this study, we aimed to evaluate the efficacy of a selected probiotic strain enriched with SeCys. To compare the effects of the Se-enriched probiotic, Se-enriched yeast, and sodium selenite on the relief of UC, we investigated their effects on intestinal inflammation and determined whether Se-enriched *L. paracasei* CCFM 1089 could be used as a new organic Se supplement.

## 2. Materials and Methods

### 2.1. Preparation of Se-Enriched Probiotic

*L. paracasei* CCFM 1089 was isolated from the feces of a healthy person in Henan, China. The method of isolating bacterial strains from feces was borrowed from Sofie [20]. It was selected as having the strongest Se enrichment ability in the preliminary experiments. The strain was inoculated into MRS medium containing 6.6 mg/L of sodium selenite and cultured at 37 °C overnight. The bacteria were collected and freeze-dried to obtain Se-enriched bacterial powder, which was stored at 4 °C until further study.

### 2.2. Determination of Se Concentration and Analysis of Se Form

The Se concentration in *L. paracasei* CCFM 1089 strain was determined by hydride atomic fluorescence spectrometry (AFS-8520, Beijing Haiguang Instrument Co., Ltd., Beijing, China), as reported by Li. et al. [21].

The Se forms in the strains were detected using high-performance liquid chromatography (HPLC, Ultimate3000, Thermo Fisher Scientific, Waltham, MA, USA) and inductively coupled plasma mass spectrometry (ICP-MS, NexION 350D, Perkin-Elmer, Waltham, MA, USA) according to Micaela Pescuma et al. [22]. Standard samples of SeCys (98%), methylselenocysteine (MeSeCys; 95%), SeMet (99%), sodium selenate (Na_2_SeO_4_; 98%), and sodium selenite (Na_2_SeO_3_; 98%) were purchased from Sigma-Aldrich LLC., Saint Louis, MO, USA. 

### 2.3. Animal Experiment Design

Male C57BL/6J mice (*n* = 45, 7-week-old, 22–24 g) were supplied by the Institute of Model Zoology, Nanjing University (Jiangsu, China) and kept under constant temperature (20 °C ± 2 °C) and humidity (50% ± 5%) and a 12-h light–dark cycle at the Animal Center of Jiangnan University. The protocol was approved by the Animal Ethics Committee of Jiangnan University (JN. No. 20201030c0550115[293]). The mice were randomly divided into nine groups: control, model, Se deficiency control (Se-de control), Se deficiency model (Se-de model), *L. paracasei* CCFM 1089, Se-enriched *L. paracasei* CCFM 1089 (Se-CCFM 1089), Se-enriched inactivated *L. paracasei* CCFM 1089 (Se-in CCFM 1089), Se-enriched yeast (Se-yeast), and sodium selenite (NaSeO).

**Figure 1:**
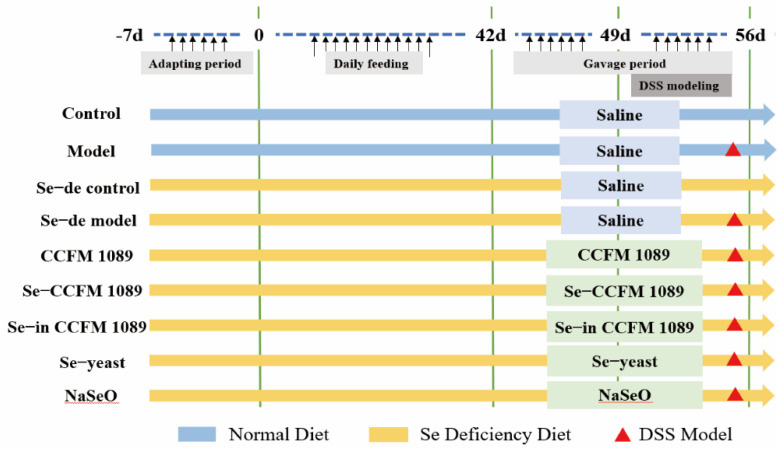
Progress of the animal experiment.

The design of the animal experiments is shown in Figure 1. In brief, the control group and the model group mice were fed a normal diet (0.2 mg/kg Se), and the remaining group mice were fed a Se-deficient diet for 6 weeks (0.02 mg/kg Se) [23,24]. Se-deficient diet was produced by Trophic Animal Feed High-tech Co. (Nantong, Jiangsu, China). Further, the mice (control and model groups) were orally administered with saline, *L. paracasei* CCFM 1089, Se-enriched *L. paracasei* CCFM 1089, Se-enriched inactive *L. paracasei* CCFM 1089, Se-enriched yeast, or sodium selenite at doses of 0.4 µg Se/d for 14 days. During this period, a Se-deficient diet was still provided to the other groups. The activity of *L. paracasei* CCFM 1089 and Se-enriched *L. paracasei* CCFM 1089 was 9.6 × 10^8^ CFU/mL. Se-enriched yeast was purchased from Angel Yeast Co. (Yichang, Hubei, China) and sodium selenite was purchased from Sigma-Aldrich LLC. Except for the control and the Se-de control group mice, the remaining mice were treated with 2.5% (*w*/*v*) DSS (MP Biomedicals LLC, Irvine, CA, USA) to induce IBD during the last week of the gavage period.

During the experimental period, the body weight, food intake, stool consistency and presence of blood in the stool of the mice were recorded daily, occult blood was measured using Occult Blood Test Kit [25] (Zhuhai Beisuo Biotechnology Co. Ltd., Zhuhai, China), and the health and behavior of the mice was observed regularly. At the end of the experiment, mice were narcotized and sacrificed. The blood of the mice was centrifuged, and the serum was stored at −80 °C until further analysis. The liver, colon, and ileum tissues were also collected and stored at −80 °C until further use.

### 2.4. Assessment of the Severity of Colitis

Changes in the body weight and disease activity index (DAI) of mice were measured daily during the entire period, according to the previous literature [26]. Colon length was measured after the experiment.

The colon tissues were soaked in 4% paraformaldehyde overnight, dehydrated, embedded, sliced, stained with hematoxylin and eosin (H & E), and visualized under the Pannormic MIDI (3DHistech Ltd., Budapest, Hungary). The pathological scores were evaluated using the histological scoring system reported in previous studies [27,28].

### 2.5. Examination of Oxidative Activity and Level of Cytokines in Colon Tissue via Assays

Colon tissues were washed twice with PBS buffer solution and homogenized with tissue lysis buffer solution. The supernatant was collected to detect the antioxidant activity of the colon. Malondialdehyde (MDA), reactive oxygen species (ROS), glutathione peroxidase (GSH-Px), and superoxide dismutase (SOD) levels were analyzed according to the manufacturer’s instructions on the respective kits (Nanjing Jiancheng Biotechnology Institute, Nanjing, Jiangsu, China). Protein concentration was measured using a BCA protein assay kit (Beyotime Biotechnology, Shanghai, China). The activities of SOD, catalase (CAT), and GSH-Px are presented as pictograms of U/mg colon protein. The concentrations of TNF-α and interleukin IL-10, IL-1β, and IL-6 in colon tissues were measured using ELISA kits (R&D, Farmington Hills, MI, USA), the sensitivity of TNF-α was 7.21 pg/mL, IL-10 was 5.22 pg/mL, IL-1β was 4.8 pg/mL, and IL-6 was 1.8 pg/mL.

### 2.6. Extraction of Total RNA of Colon and Quantitative Polymerase Chain Reaction (qPCR)

Total RNA was extracted from the colon of mice using TRIzol [29]. After determining the total RNA concentration by the NanoDrop 2000c (Thermo Scientific, Bannockburn, IL, USA), cDNA was synthesized using an equal starting concentration (1 μg) for reverse transcription with HiScript III RT SuperMix for qPCR (Vazyme Biotechnology Co., Jiangsu, China). The qPCR reaction conditions were employed: ① 95 °C for 2 min; ② 95 °C for 15 s, 60 °C for 30 s, 40 cycles; ③ 95 °C for 15 s; ④ 60 °C for 1 min; ⑤ 95 °C for 15 s. β-actin was used as an internal control. Amplified primers were synthesized by Sangon Bioengineering Co., Ltd., (Shanghai, China), and their sequences are listed in Table 1 [30].

### 2.7. Determination of Serum Biochemical Indices

The activities of high-density lipoprotein cholesterol (HDL), alanine aminotransferase (ALT), aspartate aminotransferase (AST), and albumin (ALB) in the serum of mice were detected using a Mindray automatic biochemical analyzer (Mindray-BS 480; Mindray, Shenzhen, China).

### 2.8. Measurement of Se Concentration in Liver and Ileum

Liver and ileum samples were digested in nitric acid for 12 h at the room temperature, and digestions were performed at 120 °C for 2 h, then added hydrochloric acid. After cooling, the samples were diluted with 5% hydrochloric acid and the Se concentration was measured by hydride atomic fluorescence spectrometry (AFS-8520, Beijing Haiguang Instrument Co., Ltd., Beijing, China).

### 2.9. Statistical Analysis

The data are expressed as mean ± standard deviation (SD). Statistical analyses were performed using Origin software (OriginLab, Northampton, MA, USA). Statistical comparisons between different groups were performed using one-way analysis of variance (ANOVA) and Tukey’s post-hoc test with SPSS (v23.0, SPSS Inc., Chicago, IL, USA). The results were considered statistically significant at *p* < 0.05.

## 3. Results

### 3.1. Data of Se-Enriched Products

As shown in Table 2, the Se level of the freeze-dried powder of Se-enriched *L. paracasei* CCFM 1089 was 334.9 mg/L, and its viable count was 2.65 × 10^10^ CFU/g. The number of viable bacteria in the freeze-dried powder of *L. paracasei* CCFM 1089 was 2.71 × 10^11^ CFU/g.

**Figure 2:**
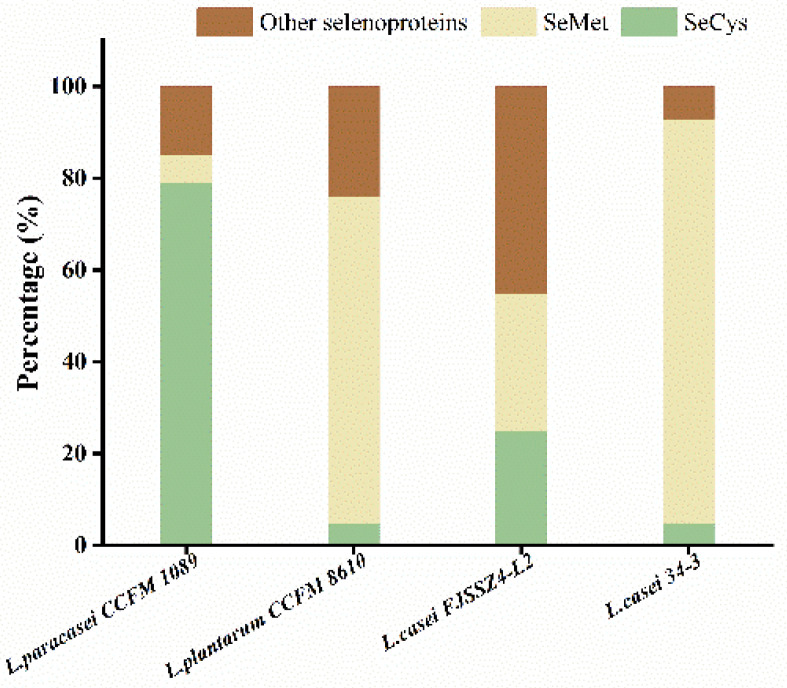
Results of Selenium (Se) form analysis. The four strains compared are *Lactobacillus paracasei* CCFM 1089, *Lactobacillus plantarum* CCFM 8610, *Lactobacillus casei* FJSSZ4-L2, and *Lactobacillus casei* 34-3, respectively.

As shown in Figure 2, the values were averaged by repeating the measurements three times. The SeCys content in Se-enriched *L. paracasei* CCFM 1089 was 79% of organic Se, which was much higher compared with than that of other strains in the previous experiment. As shown in Table 3, the Se level of the Se-enriched yeast was 2000 mg/L, and the SeMet content was 65.3%.

### 3.2. Se-Enriched Products Affect the Symptoms of Colitis in Mice

**Figure 3:**
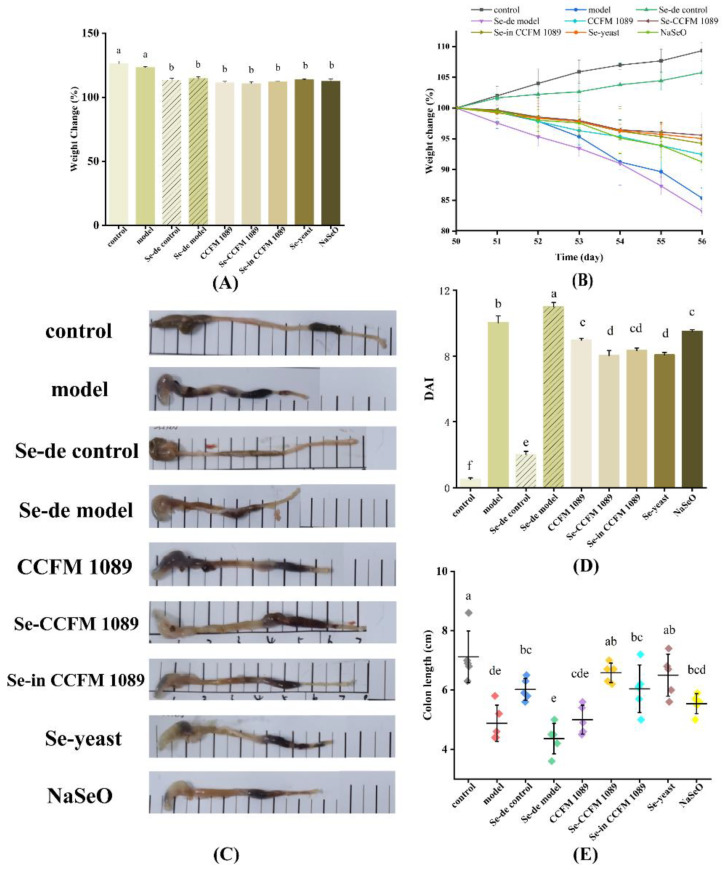
Symptoms of DSS-induced colitis. (**A**) Body weight before DSS treatment; (**B**) body weight during DSS treatment; (**C**) pictures of mice colons; (**D**) disease activity index (DAI); (**E**) colon length. Graphs show mean ± SD; *n* = 5. Significant differences are indicated by different superscript letters (*p* < 0.05), and the same superscript letters above the bars indicate that the differences between the values are not statistically significant (*p* > 0.05).

Due to Se-deficient feeding, the mice in the Se-deficient groups grew slowly compared to the control groups (Figure 3A), and their growth was significantly different from that of mice in normal-diet groups. During DSS treatment, the mice lost a significant amount of weight (Figure 3B) and their DAI increased (Figure 3D). However, Se treatment alleviated DSS symptoms to some extent. Mice in the Se groups had normal activity, with anorectic and vertical hair, whereas mice in model groups were lethargic, had reduced activity, disheveled hair, and unshaped feces. The rate of weight loss of mice in the organic Se groups was relatively slow, at around 4%, whereas that of mice in the NaSeO group was 9%. The analysis showed that treatment with different forms of Se had different effects on DAI. Se-enriched *L. paracasei* CCFM 1089 group mice experienced the strongest relief effect, with a DAI of 8.23 ± 0.31 on the seventh day. Compared with the mice in the model group, Se-enriched *L. paracasei* CCFM 1089 group mice displayed a 46.76% decrease in the DAI. The remission effects in the mice in the three organic Se groups were similar. However, the DAI of the NaSeO group mice was 9.50 ± 0.10, showing the smallest decrease.

The effects of different Se-enriched products on the gut of mice were evaluated. The colon length of the control group mice was 7.12 ± 0.87 cm (Figure 3E), the colon was normal and red, and the feces were granular. The colon length of the model group mice was 4.88 ± 0.61 cm, the colon was dark red, and blood was detected in the stool (Figure 3C). An analysis of the data revealed that *L. paracasei* CCFM 1089 had the worst effect on maintaining the colon length of mice, with a shortening rate of 25.0%. The colons of the mice in organic Se groups were longer than those of mice in the inorganic group. Furthermore, there was little difference in effect among the mice in the three organic Se groups, with the Se-enriched *L. paracasei* CCFM 1089 treatment showing the best effect with a numerical comparison, and a shortening rate of 5.6%.

### 3.3. Se-Enriched Products Ameliorate Inflammatory Injury Caused by DSS in Mice

**Figure 4:**
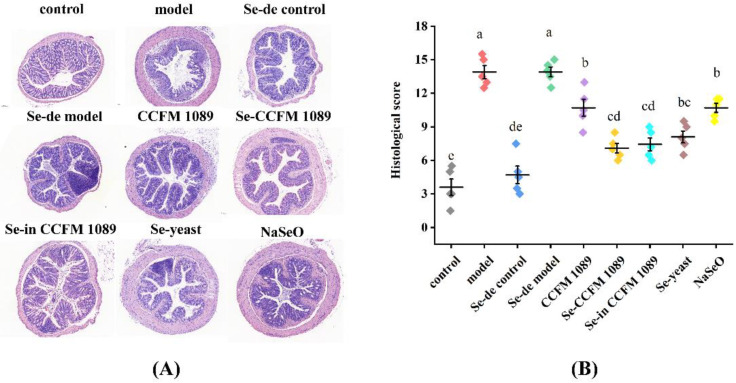
Effects of different Se forms on the histological injury in the colon with colitis. (**A**) Histological examination (the scale bar is 100 μm). (**B**) Colon tissue injury scores. Graphs show mean ± SD; *n* = 5. Significant differences are indicated by different superscript letters (*p* < 0.05), and the same superscript letters above the bars indicate that the differences between the values are not statistically significant (*p* > 0.05).

Histopathological examination was performed to evaluate the effects of different Se-enriched products on colon histopathological injury in DSS-induced mice. As shown in Figure 4A, the colons of the control group mice had intact mucous membranes and neat villi with healthy crypt structures. However, the model group mice showed serious colonic mucosal damage, such as disappearance of the mucosal muscularis, severe infiltration of inflammatory cells and colonic crypt loss. The colon injury score of the model group was 13.90 ± 1.29 (Figure 4B), which was significantly higher than that of the control group (3.60 ± 1.64). Organic Se intervention reduced the infiltration of inflammatory factors into the colon, and the crypt structure became intact. Colon injury scores in the organic Se groups were significantly lower. The score of Se-enriched *L. paracasei* CCFM 1089 was 7.10 ± 0.96, the score of Se-enriched inactivated *L. paracasei* CCFM 1089 was 7.44 ± 1.28, and the score of Se-enriched yeast was 8.10 ± 1.19. Only treatment with *L. paracasei* CCFM 1089 or inorganic Se provided minimal relief for colitis. The tissue injury score of the CCFM 1089 group and the NaSeO group was 10.92 ± 1.59 and 10.70 ± 0.91, respectively.

### 3.4. Se Concentration in Liver and Ileum

**Figure 5:**
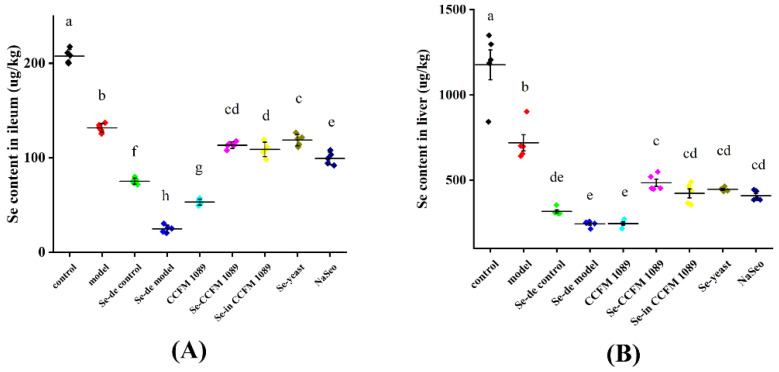
Se concentration in liver and ileum of mice. (**A**) Se content in ileum; (**B**) Se content in liver. Graphs show mean ± SD; *n* = 5. Significant differences are indicated by different superscript letters (*p* < 0.05), and the same superscript letters above the bars indicate that the differences between the values are not statistically significant (*p* > 0.05).

To observe the absorption and metabolism of different Se forms in mice, the Se concentrations in the liver and ileum of mice were determined using hydride atomic fluorescence spectrometry. Dietary Se deficiency could significantly lower the Se levels in the organs, which were improved to varying degrees by Se supplementation. As shown in Figure 5A, the Se-enriched yeast group mice had the highest Se concentration in the ileum, followed by the Se-enriched *L. paracasei* CCFM 1089 group mice. There was no significant difference between the Se concentrations in the ileum of the mice in these two groups. Furthermore, the Se concentration in the ileum of the *L. paracasei* CCFM 1089 group mice was the lowest. As shown in Figure 5B, the highest Se concentration was found in the liver of the Se-enriched *L. paracasei* CCFM 1089 group mice, and the lowest Se concentration was found in the liver of the *L. paracasei* CCFM 1089 group mice. These results suggested that mice differ in the absorption and metabolism of different Se sources.

### 3.5. Se-Enriched Products Reduce Oxidative Stress in Mice

**Figure 6:**
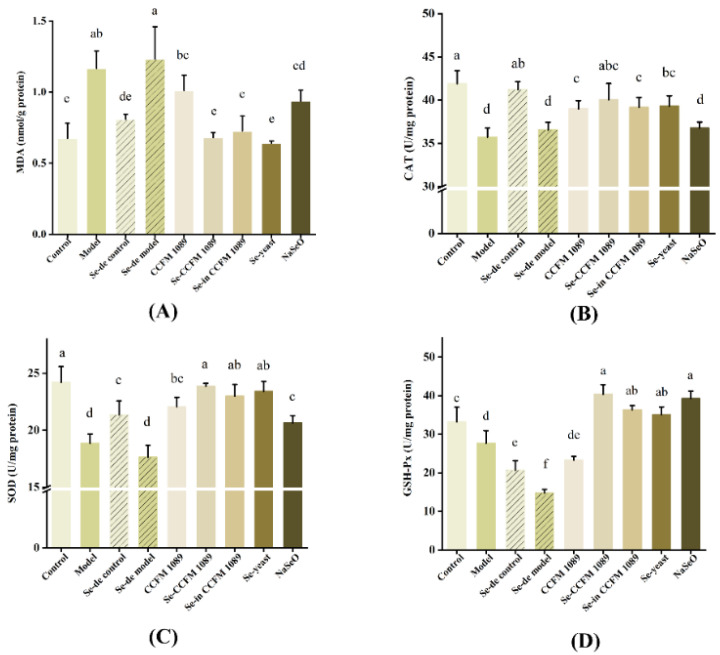
Effects of different Se products on the activity of oxidative enzymes in colon. (**A**) Malon dialdehyde (MDA) levels; (**B**) Catalase (CAT) levels; (**C**) Superoxide dismutase (SOD) levels; (**D**) Glutathione peroxidase (GSH-Px) levels. Graphs show mean ± SD; *n* = 5. Significant differences are indicated by different superscript letters (*p* < 0.05), and the same superscript letters above the bars indicate that the differences between the values are not statistically significant (*p* > 0.05).

As shown in Figure 6, compared with the model group mice, CAT and SOD activity in Se-enriched *L. paracasei* CCFM 1089 group mice significantly increased, with a 1.12-fold increase in CAT activity and a 1.27-fold increase in SOD activity. The effects on the SOD and CAT activities of mice in the Se-enriched yeast group and the Se-enriched inactivated *L. paracasei* CCFM 1089 group were similar to those in the Se-enriched *L. paracasei* CCFM 1089 group. Meanwhile, treatments with organic Se reduced the MDA levels in mice; the Se-enriched yeast group mice had the lowest MDA concentration, followed by the Se-enriched *L. paracasei* CCFM 1089 group mice. In contrast, the results showed that the alleviation effect of sodium selenite and *L. paracasei* CCFM 1089 was not ideal. In addition, by measuring the GSH-Px content, it was found that Se deficiency and colitis can decrease enzyme activity. After treatment with Se supplementation, GSH-Px activity significantly increased. The results showed that the enzyme activities of mice in Se-enriched *L. paracasei* CCFM 1089 and NaSeO groups were the highest, followed by Se-enriched inactived *L. paracasei* CCFM 1089 group and Se-enriched yeast group mice. There was not much difference between the four groups.

### 3.6. Se-Enriched Products Regulate Inflammatory Cytokines in Mice

**Figure 7:**
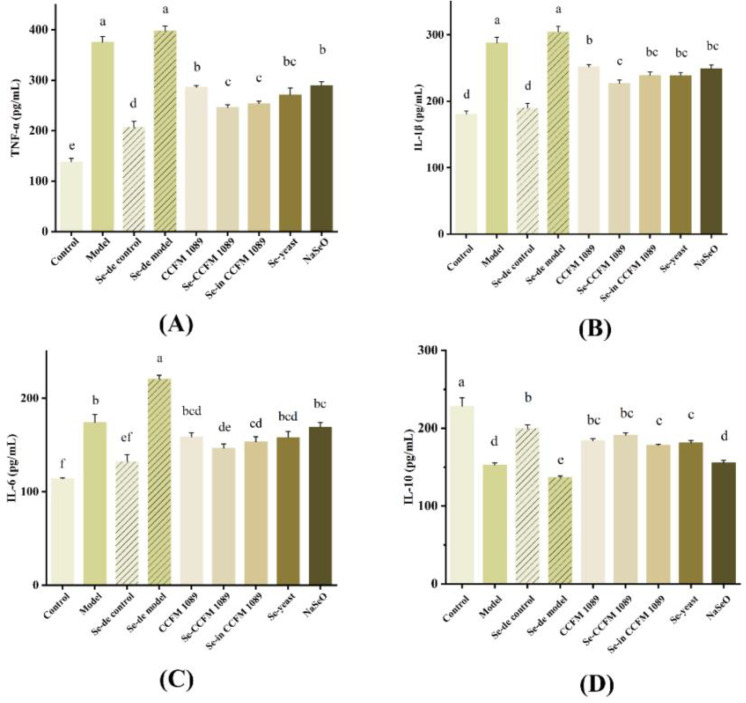
Effects of different Se products on interleukin concentration in colon. (**A**)TNF-α levels; (**B**) IL-1β levels; (**C**) IL-6 levels; (**D**) IL-10 levels. Graphs show mean ± SD; *n* = 5. Significant differences are indicated by different superscript letters (*p* < 0.05), and the same superscript letters above the bars indicate that the differences between the values are not statistically significant (*p* > 0.05).

As shown in Figure 7, the levels of pro-inflammatory cytokines (TNF-α, IL-1β, and IL-6) were significantly higher in the model group mice than in the control group mice. The mice in treatment groups had reduced levels of analysed pro-inflammatory cytokines, and the three organic Se groups were the most effective at reducing the levels of pro-inflammatory cytokines. The concentration of pro-inflammatory cytokines was the highest in the NaSeO group mice. In case of the anti-inflammatory factor IL-10 in mice, levels of Se-enriched *L. paracasei* CCFM 1089 significantly increased, followed by Se-enriched yeast, while NaSeO played an unsatisfactory role in increasing its concentration.

### 3.7. Blood Biochemical Assay

**Figure 8:**
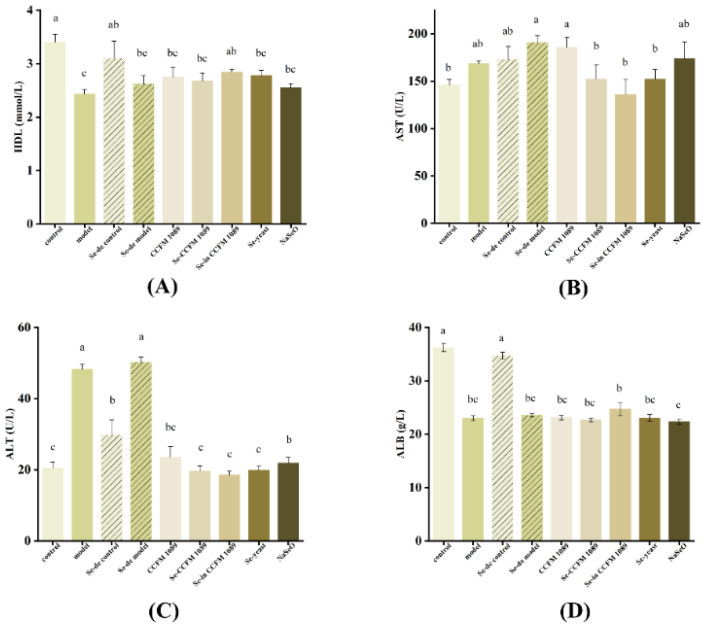
Effects of different Se products on serum biochemical indicators. (**A**) High-density lipoprotein (HDL; (**B**) aspartate aminotransferase (AST); (**C**) alanine aminotransferase (ALT); (**D**) albumin (ALB). Graphs show mean ± SD; *n* = 5. Significant differences are indicated by different superscript letters (*p* < 0.05), and the same superscript letters above the bars indicate that the differences between the values are not statistically significant (*p* > 0.05).

The effect of the treatment on the serum biochemical parameters of the mice is shown in Figure 8. HDL, AST, ALT, and ALB levels differed between the mice in the treatment groups (*p* < 0.05). After gavage of the corresponding substances, the treatment groups were almost non-differential from the model group in ALB levels; the highest level was observed in Se-enriched inactived *L. paracasei* CCFM 1089 group mice, which reached 26 g/L. The best performance with regard to the remaining three indicators was also observed in Se-enriched inactived *L. paracasei* CCFM 1089 group mice, in which the HDL content significantly increased and the AST and ALT content decreased. The effect of Se-enriched yeast and Se-enriched *L. paracasei* CCFM 1089 is close to Se-enriched inactived *L. paracasei* CCFM 1089. In contrast, the inorganic Se group mice had the lowest HDL content and the highest concentrations of AST and ALT. Among the four indexes, *L. paracasei* CCFM 1089 had the most significant effect on the ALT level, which could effectively reduce the ALT level without any significant difference from the control group.

### 3.8. Effect of Se-Enriched Products on Tight Junction Protein Expression

**Figure 9:**
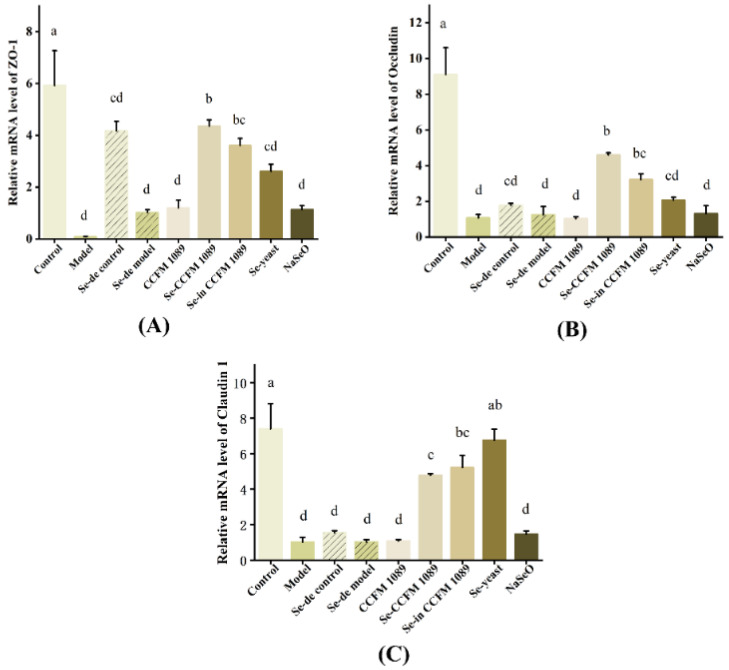
Expression of tight junction proteins ZO-1, occludin, and claudin-1 in colon. (**A**) ZO-1, (**B**) Occludin, (**C**) Claudin-1. Graphs show mean ± SD; *n* = 5. Significant differences are indicated by different superscript letters (*p* < 0.05), and the same superscript letters above the bars indicate that the differences between the values are not statistically significant (*p* > 0.05).

To compare the alleviating effects of different Se supplements on the intestinal tract of mice, the comparative gene expression of selected tight junction proteins was analyzed and is shown in Figure 9. Except for organic Se, the effect of the other treatments was negligible. The expression of claudin-1 protein in the Se-enriched *L. paracasei* CCFM 1089 and Se-enriched inactivated *L. paracasei* CCFM 1089 groups was lower than that in the Se-enriched yeast group. However, the expression of occludin and ZO-1 in the Se-enriched *L. paracasei* CCFM 1089 and Se-enriched inactivated *L. paracasei* CCFM 1089 groups was the highest in all treatment groups, and extremely close to that found in mice in the control groups.

## 4. Discussion

Previous literature [31] has verified the protective effect of Se on DSS-induced colitis, but most of the experiments were carried out when Se levels were relatively abundant, and comparisons of different forms of organic Se are few. The oral administration of Se at normal levels may induce toxicity in mice. Therefore, in this experiment, we used DSS to induce Se deficiency and compared the effect of different Se supplements on colitis relief. This experiment showed that Se-deficient mice had more severe symptoms than normal mice, which is consistent with the results of Krehl S [32]. Organic Se was found to be superior to inorganic Se in alleviating UC; moreover, different organic Se had different alleviating effects. Therefore, organic Se should be preferred over other forms for Se supplementation. Moreover, the selection of high-quality organic Se is becoming a popular research direction.

The metabolic pathways of dietary Se absorption and metabolism are significantly different [33,34]. Se is absorbed in all parts of the small intestine, with the liver being the center of Se metabolism. The absorption rate of inorganic Se is lower than that of organic Se and is generally above 50%, whereas that of organic Se can reach 90% [35]. After data analysis, it was observed that the CCFM 1089 group mice displayed little remission effect. Moreover, Se content in the NaSeO group mice was lower than that in the organic Se groups mice. Although the differences among the organic Se groups were not significant, the Se uptake of mice in Se-enriched yeast group was numerically analyzed to be higher than that in Se-enriched *L. paracasei* CCFM 1089 and Se-enriched inactive *L. paracasei* CCFM 1089 groups. The reason for this is not clear, but we confirmed that Se uptake is related to its form. Regarding the metabolism of Se, Se-enriched *L. paracasei* CCFM 1089 group mice deposited more Se than the Se-enriched yeast group mice, which may also be related to the diverse forms of Se. During absorption by the ileum, sodium selenite is degraded intracellularly to SeO_3_^2−^, which reacts with reduced GSH to form glutathione selenose trisulfide (Gs-Se-SG) under the action of certain enzymes to produce HSe^-^. Previous research has shown that SeMet is absorbed via intestinal methionine transporters, and enters the methionine pool in the body. The other way of SeMet is absorbed is via the metabolism, which occurs mostly in the liver. SeCys is produced via the methionine cycle and transsulfuration pathway, and then transported into the dynamic Se pool to participate in metabolic reactions. SeCys degrades into HSe^−^, which synthesizes SePhp with the participation of ATP and is involved in the synthesis of selenoprotein, thus exerting its antioxidant and immune effects. This may be the reason for the high Se concentration in the liver of Se-enriched CCFM 1089 group mice. The concentration of SeCys in tissues is very low, suggesting that highly reactive SeCys is kept at very low concentrations, whereas the less reactive SeMet is metabolized as if it were methionine. SeMet absorption and metabolism is competitively inhibited by methionine, but the absorption of SeCys is not competitively inhibited by cysteine. SeMet and SeCys are absorbed by extracellular pathways mediated by transporters, which are largely shared with sulfur-containing analogues. SeMet is absorbed through Na+ dependent processes, but the identity and affinity of the transporter remains to be determined. Interestingly, the Se-enriched inactive CCFM 1089 group mice deposited less Se than the Se-enriched yeast group mice. We hypothesized that probiotics may play a synergistic role in SeCys, but this remains to be studied.

The health of animals is related to their antioxidant capacity [36]. The oxidation system includes excessive ROS that induce oxidative stress. Free radicals act on lipids to produce peroxidation reactions, thereby attacking cell membrane lipids and simultaneously causing damage to colonic mucosa [37]. The pathogenesis of IBD is also associated with oxygen-radical-induced lipid peroxidation [38]. SOD is the main antioxidant enzyme that can scavenge superoxide anion radicals, and the end product of lipid peroxidation [39]. It reflects the degree of lipid peroxidation in the body and indirectly reflects the degree of cell damage. CAT can continuously remove the main oxidation products generated in the body and maintain the stability of the cell membrane [40]. In this study, the effect of *L. paracasei* CCFM 1089 and NaSeO on the levels of MDA, CAT, and SOD in mice was not as strong as that of organic Se. Of all the treatment groups, mice in the Se-enriched yeast group had the lowest MDA levels, and mice in the Se-enriched *L. paracasei* CCFM 1089 group had the highest CAT and SOD levels. Thus, it is believed that different Se forms have different response pathways to inhibit oxidative stress.

Notably, the mice in the treatment groups showed higher GSH-Px activity, especially those in the Se-enriched *L. paracasei* CCFM 1089, Se-enriched inactivated *L. paracasei* CCFM 1089, and NaSeO groups. GSH-Px is an important member of the antioxidant system that can metabolize intracellular ROS and maintain cellular homeostasis. It is a type of selenase, in which the active center is SeCys. GSH-Px activity can reflect the Se level of the body, and Se is a component of the GSH-Px system [41]. Previous research has shown that LTB4 is a chemokine that induces neutrophils into inflammatory areas. Peroxide mediates the production of active LTB4. GSH-Px can remove peroxide and inhibit the production of LTB4. Arachidonic acid can be converted into the precursor of LTB4 under the catalytic activity of 5- and 15-lipoxygenase, and selenase can inhibit the production of LTB4 by inhibiting the activities of GSH-Px. This may be the reason for the high antioxidant capacity of Se-enriched *L. paracasei* CCFM 1089. Interestingly, Beilstein and Whanger [42] found, in their early study, that the main form of Se was SeCys in the tissues of rats fed with sodium selenite using isotope tracer 75Se, whereas SeMet was present in the hemoglobin of rats fed with SeMet. Since the major form of Se in Se-enriched *L. paracasei* CCFM 1089 and Se-enriched inactivated *L. paracasei* CCFM 1089 is SeCys, we speculated that SeCys might be better than SeMet at enhancing the selenase activity. This hypothesis needs to be further confirmed in other studies.

Abnormal immune responses are also a risk factor for IBD, causing an increase in intestinal permeability and facilitating the entry of endotoxins into the body, causing immune system disorders [43]. Cytokines are mainly involved in immune and inflammatory responses. Moreover, they are partially involved in immune cell infiltration and the release of inflammatory mediators [44,45]. It has been reported that TNF-α can damage the intestinal barrier and cause intestinal damage [46]. Moreover, IL-6 and IL-1β can disrupt intestinal flora [47]. Our study found that the secretion of pro-inflammatory factors TNF-α, IL-1β, and IL-6 was inhibited in the mice in the treatment groups. Among them, the best inhibition effect was observed in the mice in the three organic Se groups. The expression levels of the three pro-inflammatory factors in mice were downregulated in the three organic Se groups, and were significantly different from those in the model group. DSS treatment has been shown in previous studies to activate the NF-κB pathway, which causes the release of inflammatory cytokines TNF-α and IL-1β, aggravating the development of IBD [48,49]. Previous studies have shown that the levels of TNF-α and IL-1β decreased in the NLRP3^−/−^mice with colitis, indicating that NLRP3 could regulate the release of inflammatory cytokines [50]. Therefore, the possible anti-inflammatory action of Se on colitis may be related to the inhibition of NF-κB and NLRP3 pathways. Se-enriched *L. paracasei* CCFM 1089 may inhibit the activation of macrophages. IL-10 is considered an important anti-inflammatory factor in organisms [51], which can maintain tissue integrity and promote the healing of inflammation-induced injuries. Among the mice in the treatment groups, Se-enriched *L. paracasei* CCFM 1089 group mice had the highest IL-10 concentration, followed by the mice in Se-enriched yeast and Se-enriched inactived *L. paracasei* CCFM 1089 groups. We assumed that this was related to the fact that organic Se could enhance intestinal thickness or inhibit the destruction of intestinal mucosa caused by inflammation; therefore, we compared the mRNA expression of Tight junction (TJ) proteins in the intestinal tract of mice.

TJ have been proven to maintain cell polarity and intestinal barrier function. ZO-1, occludin and claudin-1 are the main proteins that maintain the mechanical barrier and permeability of the intestinal mucosa mucosal [52]. Free radicals can cause damage to the intestinal mucosa and reduce intestinal permeability. In this study, we also found that Se deficiency caused serious damage to the intestinal mucosa by lowering the expression levels of occludin and claudin-1. It is obvious from the results that organic Se can repair the damage in intestinal mucosa; the expression of ZO-1 and occludin in the mice in Se-enriched *L. paracasei* CCFM 1089 and Se-enriched inactived *L. paracasei* CCFM 1089 groups was higher than that in Se-enriched yeast group. We hypothesized that the maintenance of intestinal barrier function differs in Se form and that the protective effect of SeCys may be better than that of SeMet. It was also found that, because of its probiotic activity, Se-enriched *L. paracasei* CCFM 1089 was more highly expressed than Se-enriched inactived *L. paracasei* CCFM 1089 of ZO-1 and occludin. This also validates the previous speculation that the combination of active probiotics and SeCys may act synergistically in the organism and is superior to their use as separate substances. It can be speculated that SeCys-enriched *L. paracasei* CCFM 1089 is a promising new Se supplement. As qPCR has certain limitations, the effect of the product must be further confirmed at the protein level.

Serum biochemical parameters can be used as markers of the nutritional status of animals [53]. We found that Se had an effect on the serum biochemical parameters. The highest level of HDL was found in the Se-enriched inactived CCFM 1089 group mice, followed by the Se-enriched yeast group mice. HDL can transport harmful substances to the liver via catabolic excretion. Compared to the transportation rate of SeMet, the rate of SeCys transportation of harmful substances may be slightly higher. In this study, we found that ALB content was unrelated to the Se form, which is also supported by the results of Liu et al. [54]. Elevated AST and ALT levels indicate possible kidney or liver damage; moreover, the AST and ALT levels are important indicators of liver injury. All the treatments had a good alleviating effect on the damage; organic Se had the best alleviating effect. Moreover, the levels of ALT and AST in mice in the Se-enriched inactived *L. paracasei* CCFM 1089 group were lower than those in mice in the other two organic groups. Moreover, the enzymes in the blood can reduce oxidative damage through redox reactions [55], which may also explain why the GSH-Px activity of the Se-enriched *L. paracasei* CCFM 1089 group was higher than that of the other treatment groups. Once again, this provides evidence to verify the use of Se-enriched *L. paracasei* CCFM 1089 as a new Se supplement.

In this study, *L. paracasei* CCFM 1089 was used to convert inorganic Se into organic Se through biotransformation, and then it was compared with mature Se supplements (Se-enriched yeast, sodium selenite) in terms of alleviating colitis. We also analyzed the reasons for the different effects from the perspective of Se form. This study confirmed that Se-enriched *L. paracasei* CCFM 1089 could effectively relieve colitis symptoms by regulating the redox status in mice; it could also regulate the levels of TJ proteins and immune response. Therefore, for the IBD patients, the oral administration of Se-enriched *L. paracasei* CCFM 1089 may improve the antioxidant capacity of the body by increasing Se levels in patients and protect the integrity of the intestinal mucosa, thereby relieving colitis. This speculation needs to be further investigated in clinical trials. Furthermore, our study has the limitation of not considering the effect of dose variation on the relief of colitis; more comprehensive studies are needed to make the results more widely applicable.

## 5. Conclusions

In summary, the study showed that different Se-enriched products have different alleviating effects on UC in mice due to the differences in their antioxidant capacity, as well as the different absorption and metabolic rates of different Se forms. Se-enriched *L. paracasei* CCFM 1089 effectively reduced pro-inflammatory cytokines in vivo and improved the activity of GSH-Px; it could also improve the expression of intestinal TJ proteins and maintain the integrity and length of the colon in mice. Our results suggest that Se-enriched *L. paracasei* CCFM 1089 could be confirmed to be effective in alleviating colitis and has the potential to act as a new Se supplement in the future.

## Figures and Tables

**Table 1:** Forward and Reverse Primers Sequences.

Primer	Sense (5′-3′)	Anti-Sense (5′-3′)	Gene ID
β-actin	GTGCTATGTTGCTCTAGACTTCG	ATGCCACAGGATTCCATACC	11461
Claudin-1	GCTGGGTTTCATCCTGGCTTCTC	CCTGAGCGGTCACGATGTTGTC	12737
Occludin	TTGAAAGTCCACCTCCTTACAGA	CCGGATAAAAAGAGTACGCTGG	18260
ZO-1	GCTTTAGCGAACAGAAGGAGC	TTCATTTTTCCGAGACTTCACCA	21872

**Table 2:** Viable bacteria and Se content of CCFM 1089 powder.

Strain	Number of Viable Bacteria (cfu/g)	Se Content (mg/L)
*L. paracasei* CCFM 1089	2.71 × 10^11^	-
Se-enriched *L. paracasei* CCFM 1089	2.65 × 10^10^	334.91

**Table 3:** Enrichment of organic Se in products.

Product	Se Content (mg/L)	Organic Se Content (%)	SeCys Content (%)	SeMet Content (%)
Se-enriched *L. paracasei* CCFM 1089	334.9	81.0	79.0	6.0
Se-enriched yeast	2000.0	97.0	1.1	65.3

## Data Availability

All data presented in this study are available in the main body of the manuscript.
